# 人源化与鼠源CD19 CAR-T细胞疗法治疗复发/难治急性B淋巴细胞白血病的长期随访

**DOI:** 10.3760/cma.j.issn.0253-2727.2023.10.001

**Published:** 2023-10

**Authors:** 孟仪 都, 寅嫱 张, 丹颖 廖, 薇 谢, 巍 熊, 恒 梅, 豫 胡

**Affiliations:** 1 华中科技大学同济医学院附属协和医院血液科，武汉 430022 Institute of Hematology, Union Hospital, Tongji Medical College, Huazhong University of Science and Technology, Wuhan 430022, China; 2 湖北省肿瘤疾病细胞治疗临床医学研究中心，武汉 430022 Hubei Clinical Medical Center of Cell Therapy for Neoplastic Disease, Wuhan 430022, China

**Keywords:** 嵌合抗原受体T细胞, 人源化, 急性B淋巴细胞白血病, 长期随访, Chimeric antigen receptor T cell, Humanized, B cell acute lymphoblastic leukemia, Long-term follow-up

## Abstract

**目的:**

探索人源化和鼠源CD19嵌合抗原受体T细胞（CAR-T细胞）治疗复发/难治急性B淋巴细胞白血病（B-ALL）安全性、短期及长期随访的疗效差异。

**方法:**

分析2016年5月至2023年3月于华中科技大学同济医学院附属协和医院接受CD19 CAR-T细胞治疗的80例R/R B-ALL患者的有效性和安全性，其中接受鼠源CAR-T治疗31例，人源化CAR-T治疗49例。

**结果:**

鼠源和人源化组患者发生细胞因子释放综合征（CRS）的比例分别为61.3％和65.3％，其中接受鼠源CAR-T的患者发生重症CRS的比例高于人源化CAR-T（19.4％对8.2％，*P*＝0.174），两组中分别有1例患者死于严重CRS。1～2级免疫效应细胞相关神经毒性综合征（ICANS）的发生率为12.9％和6.1％，无患者发生高级别ICANS。鼠源组和人源化组中白血病患者的总体反应率分别为74.2％和87.8％。在中位时间为10.5个月的随访期中，两组患者中位无复发生存（RFS）期均为12个月，中位总生存（OS）期均未达到。在45例基线骨髓白血病细胞负荷>20％的患者中，接受人源化CAR-T治疗的患者1年PFS率显著高于鼠源组（43.25％对33.33％，*P*＝0.027）。桥接移植是改善B-ALL患者OS（*χ*^2^＝8.017，*P*＝0.005）及RFS（*χ*^2^＝6.584，*P*＝0.010）的独立影响因素。常见高危因素（年龄、骨髓高肿瘤负荷、BCR-ABL融合基因）对长期随访结果无显著影响。3例人源化组患者多次回输后仍达完全缓解，1例鼠源组患者复发后二次回输鼠源CAR-T细胞RFS期仅1个月。

**结论:**

与接受鼠源CAR-T疗法治疗的患者相比，人源化CAR-T在不影响安全性的前提下，在高肿瘤负荷患者中显示出更持久的疗效，并有效克服免疫原性导致的CAR-T耐药，为多次复发患者提供治疗选择。

## Abstract (en)

**Objective:**

Murine CD19 chimeric antigen receptor T-cell（CAR-T）products have been approved for the treatment of refractory/relapsed（R/R）B-cell acute lymphocytic leukemia（B-ALL）; moreover, humanized products are also undergoing clinical trials. This study aimed to explore the differences in safety and short- and long-term follow-up efficacy between humanized and murine CD19 CAR-T-cells for treating relapsed and refractory B-ALL.

**Methods:**

Clinical data of 80 patients with R/R B-ALL treated with CD19-targeted CAR-T-cells at the Union Hospital of Tongji Medical College of Huazhong University of Science and Technology between May 2016 and March 2023 were analyzed, which included 31 patients with murine CAR-T and 49 with humanized products.

**Results:**

The proportion of patients with cytokine-release syndrome（CRS）in the murine and humanized groups was 63.1％ and 65.3％, respectively. Moreover, a higher proportion of patients suffered from severe CRS in the murine group than in the humanized CAR-T group（19.4％ *vs* 8.2％, *P*＝0.174）. Furthermore, one patient per group died of grade 5 CRS. The incidence of grade 1–2 immune effector cell-associated neurotoxicity syndrome（ICANS）was 12.9％ and 6.1％, respectively; severe ICANS were not observed. Among patients receiving murine CAR-T-cells, an overall response（OR）was observed in 74.2％. Conversely, the OR rate of patients receiving humanized CAR-T-cells was 87.8％. During the median follow-up time of 10.5 months, the median recurrence-free survival（RFS）of patients with murine CAR-T-cells was 12 months, which was as long as that of patients with humanized CAR-T-cells. The median overall survival（OS）were not reached in both groups. Of the 45 patients with a bone marrow burden over 20％ at baseline, humanized CAR-T therapy was associated with a significantly improved RFS（43.25％ *vs* 33.33％，*P*＝0.027）. Bridging transplantation was an independent factor in prolonging OS（*χ*^2^＝8.017, *P*＝0.005）and PFS（*χ*^2^＝6.584, *P*＝0.010）. Common risk factors, such as age, high proportion of bone marrow blasts, and BCR-ABL fusion gene expression, had no significant effect on patients' long-term follow-up outcomes. Three patients reached complete remission after reinfusion of humanized CAR-T-cells. However, one patient relapsed one month after his second infusion of murine CAR-T-cells.

**Conclusion:**

The results indicate that humanized CAR-T therapy showed durable efficacy in patients with a higher tumor burden in the bone marrow without any influence on safety. Moreover, it could overcome immunogenicity-induced CAR-T resistance, providing treatment options for patients who were not treated successfully with CAR-T therapies.

靶向CD19嵌合抗原受体T细胞（CAR-T细胞）疗法的出现，为复发/难治急性B淋巴细胞白血病（B-ALL）患者提供了新的治疗策略。多项临床研究显示，靶向CD19鼠源CAR-T细胞治疗B-ALL患者完全缓解（CR）率可达70％～90％[1]–[2]，但其在安全性和长期疗效方面仍然存在许多问题。首先是与治疗相关的不良反应，包括细胞因子释放综合征（CRS）、神经毒性、血液毒性等[3]，其中CRS是最常见的不良反应[4]。Tisagenlecleucel是首个在美国食品药品管理局获批的鼠源CAR-T产品，在治疗复发/难治B-ALL时，高达44.3％的患者发生3～4级CRS[5]。此外，尽管鼠源CAR-T细胞疗效显著，仍有30％～50％的患者在1年内复发[6]。不良反应和复发限制了鼠源CAR-T产品的广泛应用，研究人员将鼠源靶头进行人源化改造，以期提高患者的临床获益。

鼠源CAR-T的单链可变区（scFv）具有天然的免疫排斥作用，机体可通过细胞免疫和体液免疫排斥和清除CAR-T细胞。因此，CAR的免疫原性是影响CAR-T细胞长期存续或能否重复输注的重要因素[7]。配体受体结构域的人源化改造等策略可有效降低CAR的免疫原性，临床前研究证实具有完全人源化scFv的CAR能够通过逃避潜在的宿主抗CAR免疫反应获得持续的抗肿瘤活性[8]。然而，目前国内尚无对比鼠源和人源化CD19 CAR-T细胞治疗复发/难治B-ALL差异的大样本临床研究。本研究纳入接受鼠源和人源化CD19 CAR-T治疗的复发难治B-ALL患者，探究两组之间安全性、短期及长期随访的疗效差异。

## 病例与方法

1. 临床研究：本研究为回顾性队列研究，2016年5月至2023年3月，85例复发/难治B-ALL患者接受CD19 CAR-T治疗，5例患者因缺少关键临床数据予以排除，80例患者纳入临床试验。患者的诊断分型依据WHO 2016标准，疗效评估参考《中国成人急性淋巴细胞白血病诊断与治疗指南（2021版）》[9]。CR：①外周血无原始细胞，无髓外白血病；②骨髓三系造血恢复，原始细胞<5％；③中性粒细胞绝对计数（ANC）>1.0×10^9^/L；④PLT>100×10^9^/L；⑤4周内无复发。CRi：PLT≤100×10^9^/L和（或）ANC≤1.0×10^9^/L。其他应满足CR的标准。总体反应率（ORR）＝CR率+CRi率。难治定义为诱导治疗结束（一般指4周方案或Hyper-CVAD方案）未能取得CR/CRi；复发定义为已取得CR的患者外周血或骨髓又出现原始细胞（比例>5％），或影像学、活检出现髓外疾病。本研究定义CAR-T治疗高危因素包括基线时高肿瘤负荷（细胞形态学提示骨髓白血病细胞>20％）、高危基因（BCR-ABL、BCR-ABL1样、MLL、TP53、KMT2A、TCF3-HLF、IKZF1）阳性、染色体异常（低二倍体、≥5种染色体异常、21号染色体内部扩增）、中枢/睾丸浸润[9]–[10]。本研究经华中科技大学同济医学院附属协和医院伦理委员会批准，所有患者均知情同意并签署知情同意书。

2. CAR-T细胞制备及输注：鼠源CD19 CAR结构中的scFv来源于鼠FMC63单抗。人源化scFv在保留鼠源CAR的互补决定区前提下，将骨架部分用人源相似序列替代，再与共刺激域等其他序列连接，构建人源化CD19 CAR结构，经慢病毒包装、转染制备人源化CAR-T细胞（专利号ZL 2019 1 0621505.2）。采集供者或患者外周血T淋巴细胞在体外通过慢病毒载体将CAR结构片段转染进入T细胞，进行体外分离、纯化和扩增制备出转染率30％～70％的CAR-T细胞产品。预处理方案：环磷酰胺300 mg·m^−2^·d^−1^，−5～−3 d；氟达拉滨30 mg·m^−2^·d^−1^，−5～−3 d。患者在第0天回输CD19 CAR-T细胞，鼠源CAR-T细胞的中位输注量为6.5（1.5～10）×10^6^/kg，人源化CAR-T细胞的中位输注量为4（2～6）×10^6^/kg。

3. 疗效及不良反应评估：不良反应评估标准：CRS、免疫效应细胞相关神经毒性综合征（ICANS）诊断及分级参照ASTCT共识[11]；血液毒性参照美国常见不良事件评价标准（CTCAE）5.0版；CAR-T细胞治疗相关凝血病（CARAC）参照CARAC管理中国专家共识[12]；CAR相关噬血细胞综合征（CAR-HLH）参考CAR-T毒性评估及管理[13]。通过门诊或住院复查、电话随访、检索病历的方式对患者进行随访。随访时间截至2023年6月1日，中位随访时间为10.5（95％ *CI* 6～15）个月。总生存（OS）期为CAR-T细胞输注至任何原因死亡或随访终点。无复发生存（RFS）期为CAR-T治疗后获得CR至疾病复发、死亡或随访终点。

4. 统计学处理：应用Graphpad Prism 9.0进行统计学分析。分类变量以例数（百分比）描述，采用卡方检验或Fisher精确概率法进行组间比较；连续变量以中位数（范围）描述，组间比较采用秩和检验（不符合正态分布）。采用Reverse Kaplan-Meier法计算所有患者及亚组的中位随访时间；采用Kaplan-Meier法绘制生存曲线，Log-rank检验进行组间比较。双侧*P*<0.05为差异有统计学意义。

## 结果

1. 一般临床特征：共纳入80例复发/难治B-ALL患者，其中女38例，男42例，中位年龄28.5（13～67）岁。76例患者在CAR-T细胞输注前骨髓内存在肿瘤负荷，中位原始细胞比例为23.45％（0.02％～97.85％），4例患者骨髓微小残留未见原始细胞，但尚可检测到高危基因突变拷贝数。22例患者存在高危基因突变，其中16例伴BCR-ABL融合基因，3例伴MLL-AF4融合基因，2例伴TP53突变，1例同时伴MLL-AF4融合基因和TP53突变。52.5％的患者在接受CAR-T治疗前出现疾病复发，既往接受的中位治疗线数为3（1～10）线，26.3％的患者接受过造血干细胞移植，8.8％的患者接受过CAR-T治疗。31例患者回输鼠源CAR-T细胞，49例患者回输人源化CAR-T细胞，人源化组患者骨髓白血病细胞>20％比例显著高于鼠源组（*P*<0.001），而回输总细胞量则低于鼠源CAR-T细胞（*P*<0.001）（表1）。

**表1:** 接受鼠源或人源化CD19 CAR-T细胞治疗复发/难治B-ALL患者临床特征和不良反应

患者特征	总体（80例）	鼠源（31例）	人源化（49例）	统计量	*P*值
性别[例（%）]				1.568	0.210
女	38（47.5）	12（38.7）	26（53.1）		
男	42（52.5）	19（61.3）	23（46.9）		
中位年龄[岁，*M*（范围）]	28.5（13~67）	34（14~65）	25（13~67）	−2.104	0.050
复发[例（%）]	42（52.5）	18（58.1）	24（49.0）	0.628	0.428
高危因素[例（%）]	57（71.3）	15（48.4）	42（85.7）	Fisher	0.001
骨髓白血病细胞负荷>20%	45（56.3）	9（29.0）	36（73.5）	Fisher	<0.001
高危基因突变	22（27.5）	6（19.4）	16（32.7）	Fisher	0.213
中枢/睾丸浸润	2（2.5）	0（0）	2（4.1）	Fisher	0.519
既往治疗					
中位治疗线数[*M*（范围）]	3（1~10）	3（1~10）	3（1~7）	−0.940	0.331
移植[例（%）]	21（26.3）	5（16.1）	16（32.7）	Fisher	0.123
CAR-T[例（%）]	7（8.8）	1（3.2）	6（12.2）	Fisher	0.239
中位CAR-T回输量[×10^6^/kg，*M*（范围）]	4（1.5~10）	6.5（1.5~10）	4（2~6）	−6.420	<0.001
不良反应[例（%）]					
CRS	51（63.8）	19（61.3）	32（65.3）	0.133	0.716
1～2级	41（51.3）	13（41.9）	28（57.1）	1.758	0.185
3～5级	10（12.5）	6（19.4）	4（8.2）	Fisher	0.174
ICANS	7（8.8）	4（12.9）	3（6.1）	Fisher	0.421
CARAC	7（8.8）	2（6.5）	5（10.2）	Fisher	0.700
CAR-HLH	2（2.5）	0（0）	2（4.1）	Fisher	1.000
长期血液毒性^a^	3（6.7）	2（12.5）	1（3.4）	Fisher	1.000
桥接移植[例（%）]	18（22.5）	7（22.6）	11（22.4）	Fisher	1.000

注 CAR-T：嵌合抗原受体T细胞；B-ALL：急性B淋巴细胞白血病；CRS：细胞因子释放综合征；ICANS：免疫效应细胞相关神经毒性综合征；CARAC：CAR-T细胞治疗相关凝血病；CAR-HLH：CAR相关噬血细胞综合征；^a^长期血液毒性纳入45例患者，其中16例患者回输鼠源CAR-T，29例患者回输人源化CAR-T

2. 不良反应：共有51例患者发生CRS，51.3％（41/80）的患者为1～2级，8.8％（7/80）为3级，1例患者发生4级CRS，2例患者因5级CRS死亡。鼠源组和人源化组的CRS发生率分别61.3％和65.3％，其中接受鼠源CAR-T治疗的患者发生重症CRS的比例高于人源化CAR-T，但差异无统计学意义（19.4％对8.2％，Fisher，*P*＝0.174）。ICANS的发生率分别为12.9％和6.1％（Fisher，*P*＝0.421），均为1～2级，未观察到高级别ICANS发生，经激素和对症治疗后症状均好转。7例患者被诊断为CARAC，表现为血小板减少、弥散性血管内凝血（DIC）相关指标异常以及细胞因子升高，4例发生在1～2级CRS后，3例发生在3～5级CRS后。2例患者符合CAR-HLH的诊断标准，出现CRS后铁蛋白升高、三系减少伴胆红素升高、肺水肿等器官毒性，糖皮质激素、静脉注射免疫球蛋白及对症支持治疗后恢复正常。35例患者未能评估长期血液毒性，其中18例B-ALL患者在回输后3个月内行造血干细胞移植，17例患者存在未缓解、疾病进展、失访或随访时间未及等问题。其余45例患者中，6.7％（3/45）在回输后3个月仍有CTCAE 3级及以上的血液毒性。

3. 疗效：80例复发/难治B-ALL患者中，ORR为82.5％，CR率为76.3％。其中鼠源组OR率为74.2％；人源化组ORR为87.8％。截至2023年6月1日，中位随访10.5（95％ *CI* 6～15）个月，鼠源和人源化组患者的中位RFS期均为12个月，中位OS期均未达到。人源化组患者1年OS率高于鼠源组，但差异尚无统计学意义［（82.4±6.1）％对（78.3±8.8）％，*χ*^2^＝0.082，*P*＝0.774，图1］。在回输前骨髓白血病细胞负荷>20％的45例患者中，人源化组患者的1年OS和RFS率均高于鼠源组［（86.7±6.3）％对（59.3±25.2）％；（43.2±10.9）％对（33.3±27.2）％，两组间RFS差异有统计学意义（*P*＝0.027，图2）。

**图1:**
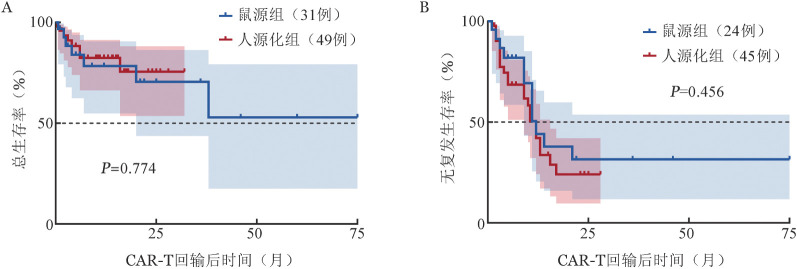
复发/难治B-ALL患者CD19 CAR-T细胞治疗后总生存（A）及无复发生存（B）曲线 CAR-T：嵌合抗原受体T细胞；B-ALL：急性B淋巴细胞白血病

**图2:**
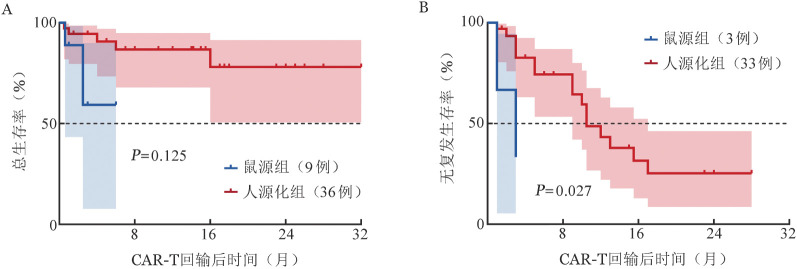
回输前骨髓白血病细胞负荷>20%复发/难治B-ALL患者CD19 CAR-T细胞输注后总生存（A）及无复发生存（B）曲线 CAR-T：嵌合抗原受体T细胞；B-ALL：急性B淋巴细胞白血病

4. 高危因素和多次回输：对影响白血病患者OS和PFS的因素进行单因素分析。桥接移植是改善患者1年OS（*χ*^2^＝8.017，*P*＝0.005）及1年RFS（*χ*^2^＝6.584，*P*＝0.010）的独立影响因素。而常见高危因素（年龄、回输前骨髓高白血病细胞负荷、BCR-ABL融合基因）对患者的OS和RFS无显著影响（表2）。

**表2:** 接受鼠源或人源化CD19 CAR-T细胞治疗复发/难治B-ALL患者1年总生存（OS）及无复发生存（PFS）的影响因素

预后因素	例数	1年OS率（%）	*χ2*	*P*值	1年PFS率（%）	*χ2*	*P*值
性别			1.033	0.309		0.701	0.402
男	42	84.88			49.35		
女	38	76.70			31.53		
年龄			0.404	0.525		0.132	0.716
≥30岁	38	82.94			44.34		
<30岁	42	79.38			41.97		
白血病细胞负荷			0.350	0.556		0.625	0.429
≥20%	45	83.14			41.49		
<20%	35	79.46			44.82		
BCR-ABL融合基因是否阳性			0.004	0.947		0.068	0.795
是	16	86.15			48.95		
否	64	70.58			50.86		
是否桥接移植			8.017	0.005		6.584	0.010
是	18	100.00			66.41		
否	62	73.16			31.83		

注 CAR-T：嵌合抗原受体T细胞；B-ALL：急性B淋巴细胞白血病

7例患者既往在本院接受过CD19 CAR-T细胞治疗。1例鼠源组患者复发后二次回输鼠源CAR-T细胞后于第14天达到CR，然而其在回输后1个月内因疾病进展死亡，体内未能检测到持续存在的CAR-T细胞。4例人源化患者二次回输后仍达CR，中位RFS期为3（1～9）个月。其中1例在接受CAR-T治疗前基线骨髓微小残留为72.4％，回输人源化CAR-T后3周内达到CR，于回输后9个月髓外复发，病理检查示CD19阳性，经二次回输后仍达CR，并实现4个月无病生存。后又因复发分别行第三和第四次CAR-T回输，最终因阴性复发退组。

## 讨论

本研究结果仍支持鼠源CD19 CAR-T细胞疗法对复发/难治B-ALL患者有效。然而，高级别CRS发生率和CAR-T存续性不足已成为CAR-T细胞治疗后长期生存的主要挑战。研究表明，鼠源CAR结构中的单链可变片段中的抗原表位可以引起HLA限制性T细胞介导的免疫反应[14]。而通过改变CAR的框架或非互补决定区域使用人源化抗体片段可减低其免疫原性，从而使CAR-T细胞治疗过程中细胞因子的释放减少并且抗肿瘤活性增强[15]。因此，人源化CAR-T细胞有可能成为高肿瘤负荷、预后不良和CAR-T回输后复发患者的治疗选择[16]。特别是对于鼠源CAR-T治疗达到CR后复发的患者，仍可获益于人源化CAR-T细胞[17]。在多发性骨髓瘤患者中，全人源BCMA靶向CAR-T 1/2期注册临床研究（NCT05066646）结果显示，纳入103例患者中，12例既往接受过CAR-T治疗，ORR仍高达96％。这表明人源化或全人源细胞可以作为鼠源CAR-T细胞治疗无效或缓解后复发的有效治疗手段。本研究中，人源化CAR-T组患者的ORR优于鼠源组，但差异无统计学意义；其在骨髓高白血病细胞负荷患者的长期缓解方面显著优于鼠源组，可能与抗CAR反应降低、CAR-T长期续存相关。

CRS是CAR-T细胞回输后最常见的不良反应，是由全身免疫细胞过度激活和增殖引起的全身炎症反应综合征[4]。研究表明CRS等级与患者基线肿瘤负荷相关，本研究中接受人源化CAR-T细胞的患者白血病细胞负荷显著高于鼠源组，尽管差异无统计学意义，但人源化CAR-T细胞在高白血病细胞负荷患者中引发3～5级CRS反应的比例低于鼠源组，可能与人源化CAR结构亲和力更强、免疫原性较低相关。此外，因鼠源CAR-T临床研究开展较早，细胞制备工艺尚不完善，CAR转染率较低，所以鼠源组的高回输剂量未导致两组间CRS发生率的差异。人源化CAR-T细胞在提升安全性的同时，通过减少CRS相关治疗避免对长期生存的影响。早期常规剂量使用糖皮质激素治疗CRS不会影响临床疗效，但长时间或大剂量使用可能会抑制患者体内CAR-T细胞的扩增及存续[18]，甚至缩短患者的PFS和OS期[19]。鼠源组患者高危CRS治疗过程中激素的使用与鼠源CAR-T细胞天然的免疫原性共同作用，可能进一步阻碍患者实现长期缓解。其他常见不良反应，如神经毒性、血液毒性和HLH在本研究中的发生比例均较低，对患者预后未造成显著影响。

接受CD19 CAR-T治疗患者的1年复发率为30％～50％[20]，CAR-T细胞在体内不能长时间存续是导致阳性复发的主要原因。人源化抗体修饰通过降低免疫原性来延长CAR-T细胞的持久性，可以在一定程度上提高治疗效果。美国费城儿童医院的临床研究中，74例接受人源化CAR-T治疗的复发/难治B-ALL患者两年的无复发率高达74％[16]。CAR-T回输有效患者可通过巩固治疗进一步延长获益。本研究中18例B-ALL患者缓解后行巩固性造血干细胞移植，患者1年的OS率达到100％，RFS率达到66％，明显优于未桥接移植组，与本中心已发表研究结论一致[21]。国内一项探究人源化靶向CD19 CAR-T细胞有效性及安全性的研究中，共纳入41例患者，5例达CR患者未接受巩固性治疗，均出现阴性复发[22]。CAR-T治疗后复发患者可再次回输人源化CAR-T细胞，获得一定时间的持续缓解[23]。随着越来越多CAR-T产品上市，如何通过产品优化和方案选择最大限度地延长患者的OS期，需要在更大的样本量、更长随访时间的前瞻性临床试验中加以探索。

当然，本研究具有一定局限性：由于鼠源CAR-T临床研究开展较早，存在患者回输剂量差异较大、未规范化CRS等不良反应后治疗方案等问题，导致队列内及队列间存在异质性。

本研究结果表明人源化CD19 CAR-T疗法在治疗复发/难治B-ALL中具有更好的长期疗效，并为CAR-T回输后复发患者提供新的治疗选择。
